# Development and validation of a Novel 3D-printed mold-based silicone model for laparoscopic duodenal stump reinforcement suture simulation training

**DOI:** 10.1186/s12909-026-09095-w

**Published:** 2026-04-13

**Authors:** Qiancheng Wang, Kuan Wang, Zhifei Wang, Guanyu Zhu

**Affiliations:** 1 Department of Gastrointestinal Surgery, Harbin Medical University Cancer Hospital, Harbin Medical University, Harbin, Heilongjiang Province China; 2 Department of Hepatobiliary and Pancreatic Surgery, Zhejiang Provincial People’s Hospital, Hangzhou Medical College, Hangzhou, Zhejiang Province China; 3 Department of Gastrointestinal Surgery, Harbin Medical University Cancer Hospital, 157 Baojian Rd, Nangang District, Harbin, Heilongjiang Province China

**Keywords:** 3D printing, Medical education, Duodenal stump fistula, Model, Simulation training

## Abstract

**Background:**

Laparoscopic duodenal stump reinforcement suture (LDSRS) is a critical yet technically challenging procedure in gastrointestinal surgery. Traditional training methods using animal models or cadavers are costly and ethically constrained. This study aims to develop and evaluate a novel, cost-effective, and safe 3D-printed mold-based silicone model for LDSRS simulation training.

**Method:**

The model was validated through a multi-phase evaluation. Ten expert laparoscopic surgeons (senior group) evaluated the model’s face and content validity using 5-point Likert scales. Twenty trainee surgeons, divided by clinical experience into intermediate (*n* = 10) and junior (*n* = 10) groups, completed ten training sessions to assess training effectiveness. Operative time and performance scores across groups were recorded and compared to assess structural validity of model.

**Results:**

Experts confirmed high face and content validity (all items > 4/5, except texture: 3.70 ± 0.82). Construct validity was strong: performance scores increased significantly with experience (junior: 17.35 ± 0.69; intermediate: 22.90 ± 0.79; senior: 33.6 ± 1.13; *p* < .001), while time decreased (junior: 22.87 ± 0.84 min; intermediate: 17.81 ± 0.32 min; senior: 4.90 ± 0.20 min; *p* < .001). Learning curves showed proficiency gains both intermediate and junior groups, with inflection points at session 6 (juniors) and session 4 (intermediates), respectively.

**Conclusion:**

The developed 3D-printed mold-based silicone model demonstrates high realism and is a valid and effective tool for LDSRS simulation training. It shows potential for improving technical skills within a simulated setting​ and may serve as a valuable component of structured surgical training curricula.

## Introduction

Duodenal stump fistula (DSF) represents a rare but serious complication after Billroth II or Roux-en-Y reconstruction following gastrectomy, with reported incidences ranging from 1.6% to 5% and mortality rates from 16% to 20% [1]. Previous research indicated that performing extra mechanical reinforcement on the duodenal stump was one of the important measures for preventing DSF [2–4]. In traditional open gastrectomy, surgeons routinely use manual sutures to reinforce the duodenal stump. However, Laparoscopic duodenal stump reinforcement suture (LDSRS) is technically challenging due to limited operating angles, difficulty in controlling knot tension, and a narrow workspace [2]. Consequently, LDSRS is predominantly performed by senior surgeons, while trainee surgeons were seldom granted operative privileges. A major challenge currently faced by trainee surgeons is the lack of sufficient opportunities for hands-on practice [5]. 

Simulation-based training in modern surgical education offers a promising method for trainee surgeons to accumulate experience and improve skills without exposing patients to risk [6]. Compared to traditional teaching methods (e.g., lectures, videos and clinical observation), simulation-based training significantly improves the overall surgical proficiency of trainee surgeons [7]. Furthermore, the surgical skills acquired through simulation training are readily transferable to real surgical settings, thereby significantly shortening the learning curve for actual surgical procedures [8, 9]. 

To date, various types of surgical simulators have been developed.Virtual reality (VR) platforms are commonly used tools for laparoscopic simulation training [8, 10–12]. Although VR simulators provide an immersive experience and help surgeons familiarize themselves with surgical procedures, they lack realistic haptic feedback and were often costly. In contrast, wet lab models (e.g., cadaveric, animal, or ex vivo organ models) offer better realism but faced challenges in widespread adoption due to non-reusability, potential infection risks, and ethical concerns [13, 14]. To overcome the limitations of existing surgical simulators, 3D-printed models have emerged as a promising alternative. As anatomically accurate, safe, and cost-effective training tools, 3D-printed models have been successfully utilized in simulation training for various surgical procedures [15–17]. However, no 3D-printed model has been specifically developed for LDSRS training.

In this study, we aimed to develop a novel, 3D-printed mold-based silicone soft-tissue model for LDSRS simulation training. A multi-phase validation study was conducted to assess its realism, educational value, and ability to discriminate between different skill levels. The model was designed to approximate the anatomical geometry and tissue consistency of the duodenal stump, thereby providing a potentially valuable platform for trainees to practice laparoscopic reinforcement sutures in a controlled setting. While this study employs traditional validity categories (face, content, construct) for clarity and clinical relevance, the collected evidence can be contextualized within contemporary frameworks for validity argument in simulation-based assessment (Messick framework) [18].

## Method

### Model design and manufacture

We designed and manufactured a silicone model using 3D-printed molds. The design data were derived from anonymized medical CT scans of a patient’s abdominal region, with approval from the Institutional Review Board (Ethics number: 2023 − 150). First, we extracted and reconstructed the 3D anatomical structures of the duodenum and surrounding tissues using the Mimics 23.0 system (Materialise, Belgium). We then imported these image data in STL format into Magic24 software for further processing, including repair, support structure design, and positioning. Next, we produced flexible molds via fused deposition modeling (FDM) 3D printing based on these data, using a silicone molding material specifically for 3D printing (Smooth-On Mold Max^®^). After printing, we performed surface treatment, removed support structures, and coated the mold cavities with petroleum jelly (Vaseline) to facilitate smooth demolding in later steps. Finally, we poured liquid silicone into the molds within a vacuum chamber and cured it at 25 °C for one hour before removal to obtain the final models. Furthermore, we iteratively adjusted the proportion of silicone oil in the liquid silicone materials based on subjective feedback from three senior laparoscopic surgeons, each with extensive experience in performing LDSRS, to replicate the tactile experience of suturing real duodenal stumps. They assessed and compared the haptic feedback (e.g., needle penetration force, tissue elasticity during suture tensioning) of the prototype models against their memory of real tissue, and refined the model until reached a consensus on the most realistic version. Further details regarding the manufacturing process of the 3D-printed mold-based silicone model have been described in our previous publications [16, 19]. 

## Components of the simulator

**Fig. 1:**
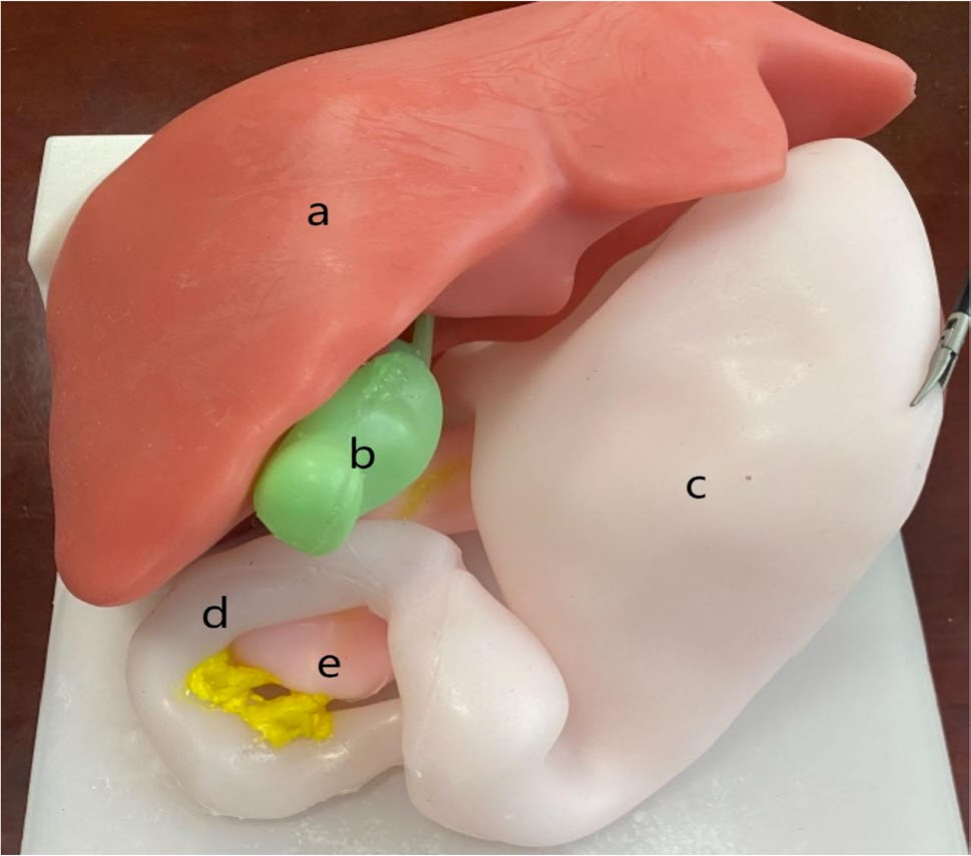
The 3D-printed model (**a**) Liver marked in red (**b**) Gallbladder marked in green (**c**) Stomach marked in white (**d**) Duodenum marked in white (**e**) Pancreas marked in pink

**Fig. 2:**
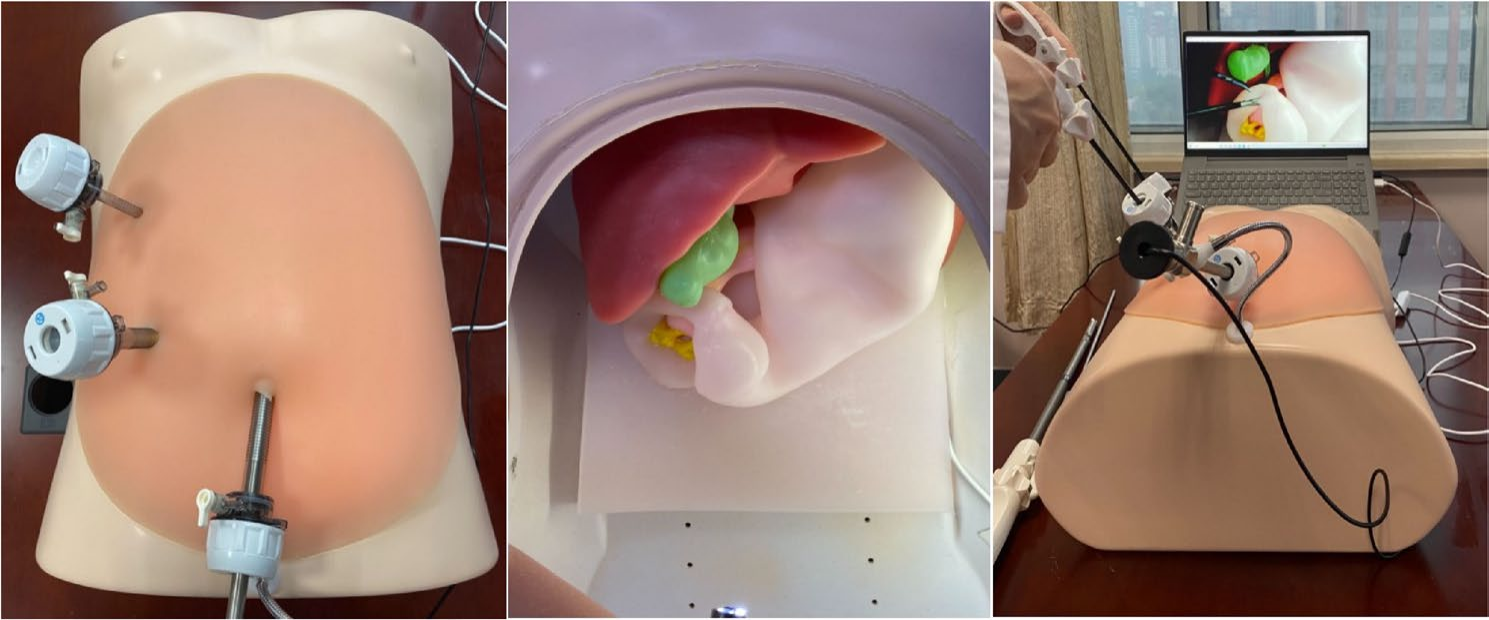
The laparoscopic training box

The simulator comprised two main components: a 3D-printed mold-based, multi-organ abdominal model and a dedicated laparoscopic training box. The multi-organ model consisted of the liver, gallbladder, pancreas, stomach, and duodenum (Fig. 1), recreating the critical anatomical relationships encountered during LDSRS. We color-coded each organ for ease of identification: the liver in red, the gallbladder in green, the pancreas in pink, and the stomach and duodenum in white. We then integrated the model into a standard laparoscopic training box, which featured a rigid silicone abdominal wall insert to simulate trocar placement and pneumoperitoneum. This setup created a high-fidelity training environment for practicing LDSRS (Fig. 2).

### Cost of model

The total direct material cost for one model was approximately 500 US dollars. The 3D printer (*Creality 3D K2*) used for mold production represents a capital cost of approximately 300 US dollars. As the printer is reusable for numerous production cycles, this cost can be amortized. We allocated the majority of the budget (400 US dollars) to the reusable liver, gallbladder, and pancreas components, which we engineered to realistically simulate the confined surgical workspace characteristic of actual LDSRS procedures. The stomach and duodenum components, costing 100 US dollars, were intentionally designed as modular and replaceable units, as they are more susceptible to wear from repeated suturing practices. This modular design strategy significantly reduces the long-term cost per training session, since only the expendable components need replacement.

### Participants

We invited ten expert laparoscopic surgeons (senior group) from our institution, each with over 10 years of experience in laparoscopic surgery and more than 300 laparoscopic gastrectomies performed as the primary operator within the past five years, to evaluate the model’s face and content validity. In addition, we recruited twenty trainee surgeons to participate in this training session from January to March 2025 to the assessment of the model’s effectiveness. We divided the trainees into two groups according to their clinical experience: ten surgical residents (intermediate group) and ten surgical interns (junior group). All interns had completed a standardized training program in the General Surgery Department at our institution between September 2021 and June 2023.

We collected demographic data and prior surgical training experiences from all twenty trainee surgeons using a structured questionnaire (see Supplementary Material 1 for details). The mean clinical experience of the trainee groups was 4.85 ± 2.28 years (Junior: 2.80 ± 0.63 years; Intermediate: 6.90 ± 1.10 years). Before the commencement of this study, all trainees had completed basic laparoscopic suturing training using silicone suturing pads. Additionally, seven surgical residents in the intermediate group had participated in more than 10 laparoscopic bowel suturing sessions using VR models, while the other three had received training with animal models. None of the trainees had prior experience performing LDSRS procedures.

### Training process

All participants underwent a standardized, simulation-based training session in LDSRS. The protocol was structured into four sequential phases:

1. Pre‑training Video Instruction: Prior to hands‑on practice, each trainee watched a pre‑recorded instructional video that detailed the procedural steps of LDSRS. The video also introduced the specific suturing technique utilized in this study—the double half purse‑string suture combined with an “8”‑shaped stitch—as previously described in our published technique report [2].
2. Expert‑led Demonstration: Following the video, an expert surgeon performed a structured, real‑time demonstration on the 3D‑printed duodenal model. The demonstration emphasized key operative maneuvers, correct application of the aforementioned suture technique, and identification of common technical errors.
3. Supervised Repetitive Practice: Trainees then performed repetitive practice on the simulation model under direct supervision. During this phase, the expert instructor was available to answer questions and provide guidance in real time.
4. Formative Feedback: After each practice attempt, the supervising instructor provided immediate, formative feedback tailored to the trainee’s performance, focusing on technique refinement and error correction.

**Fig. 3:**
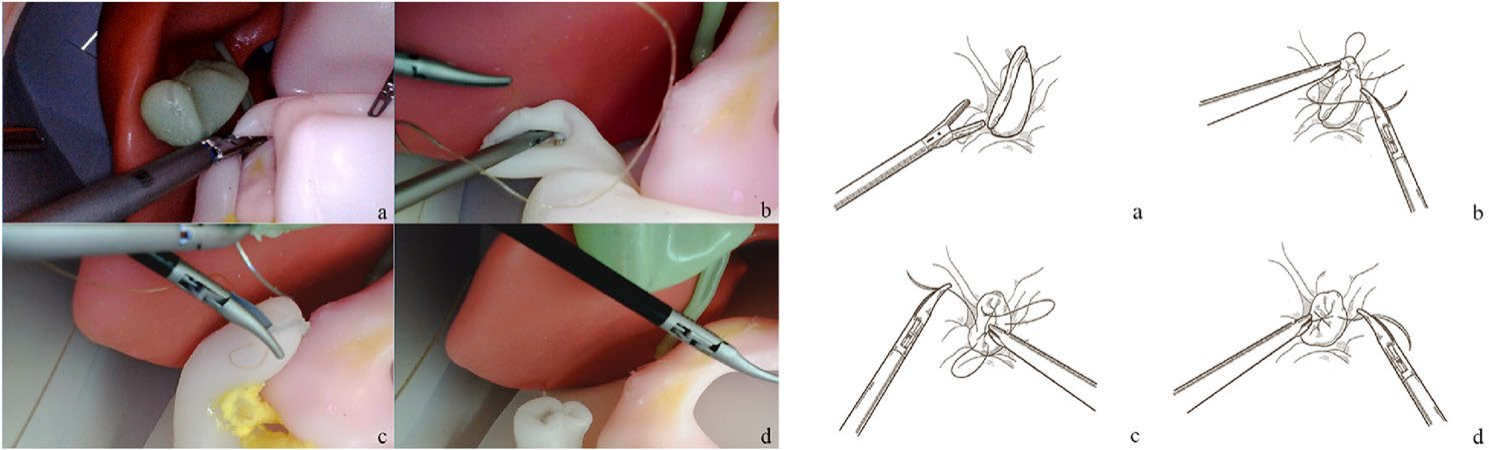
The simulation training process of LDSRS. **a** Division of the stomach and duodenum using a laparoscopic linear cutter; **b **At the upper end of the duodenal stump, a counterclockwise half purse-string suture is performed using a barbed suture; **c **At the lower end of the duodenal stump, a clockwise half purse-string suture is performed using the same barbed suture; **d **An “8” pattern suture is applied at the middle part of the duodenal stump

The simulated training process using the 3D-printed mold-based silicone model is illustrated in Fig. 3.

### Face validity and content validity of model

The validity argument for this model was structured according to Messick’s unified validity framework [18], which emphasizes multiple sources of evidence including content, response process, and relations to other variables. To assess the face and content validity of the model, we developed a 15-item multiple-choice questionnaire and administered it to the ten experts in the senior group. The first seven items evaluated the model’s visual and tactile characteristics during practical use (including overall impression, size, texture, realism, workspace, tearing tendency, and suture penetration resistance) and were designed to evaluate face validity. The remaining eight items assessed content validity by determining whether the model accurately represented the critical skills (including needle handling, suture placement, and tissue manipulation) and main purposes (including improving technical skills, enhancing confidence, accelerating the learning process, and reducing patient risk) involved in LDSRS training. The full questionnaire is available in Supplementary Material 2.

We asked the experts to rate the model using a 5-point Likert scale (1 = “strongly disagree”, 2 = “disagree”, 3 = “neutral”, 4 = “agree”, and 5 = “strongly agree”), which provided systematic quantitative data for subsequent statistical analysis [20]. This approach enabled objective measurement of expert opinions regarding the model’s characteristics and its effectiveness in representing relevant training content. To ensure the reliability of the evaluation results, we further employed Fleiss’’s Kappa coefficient (Kappa) to assess the consistency of the experts’ ratings [21]. 

To ensure the scientific rigor of the evaluation results, we developed the questionnaire based on a review of relevant literature [22, 23]. We then invited an interdisciplinary panel, comprising four certified laparoscopic training instructors, two professors from the surgical education department, and one medical statistics specialist, to review it and provide suggestions for improvement. Incorporating their suggestions and trainees’ feedback, we further revised the questionnaire to enhance its relevance and practicality, ensuring it comprehensively covered all key aspects relevant for evaluating the model’s face and content validity.

### Construct validity of model

We assessed the construct validity of the model by evaluating its ability to discriminate between surgeons with varying clinical experience [24, 25]. Upon completion of the training, we recorded the performance of all trainees using the laparoscopic simulator. Ten expert surgeons from the senior group, who were blinded to the identity and group assignment of each participant, independently reviewed surgical videos and rated operative performance using a modified Global Operative Assessment of Laparoscopic Skills (GOALS) scale.

This scale comprised seven criteria: five core metrics (depth perception, bimanual dexterity, efficiency, tissue handling, autonomy) from the original GOALS scale [26, 27], and two additional metrics (needle handling and stump burying) adapted for LDSRS-specific tasks [28]. Each metric was rated on a 5-point Likert scale (1 = “very poor”, 5 = “excellent”), with scale points corresponding to increasing levels of proficiency. We calculated the final operative score for each participant by averaging the ratings from the ten experts. A detailed description of the scale criteria is available in Supplementary Material 3. To quantitatively evaluate construct validity, we compared operative scores across the three experience groups using one-way analysis of variance (ANOVA) followed by post-hoc testing.

### Learning curve assessment

All trainees completed ten sessions of the LDSRS training program. We recorded the operative time for each trainee using the laparoscopic simulator. To evaluate changes in operational proficiency throughout the training sessions, we generated learning curves based on operation time using the cumulative sum (CUSUM) method [29, 30]. The inflection point at which the operation time stabilizes and shows no further significant changes is defined as the point of technical ‘proficiency’.

### Statistical Analysis

We performed all statistical analyses using SPSS version 28.0 (IBM Corp, Armonk, NY, USA). We expressed qualitative data as frequencies and percentages, comparing intergroup differences with the chi-square test or Fisher’s exact test as appropriate. Quantitative data are presented as mean ± standard deviation (SD), and we evaluated intergroup differences using analysis of variance (ANOVA). All tests were two-tailed, and we considered a p-value of less than 0.05 to be statistically significant.

## Results

### Baseline Characteristics

We summarized the baseline characteristics of the twenty trainees in Table 1. The cohort included nineteen males and one female, with eighteen right-hand dominant and two left-hand dominant individuals. The intermediate group was significantly older (32.20 ± 2.20 years vs. 25.90 ± 0.74 years, *P* < .001) and had more clinical experience (6.90 ± 1.10 years vs. 2.80 ± 0.63 years, *P* < .001) than the junior group. We also observed statistically significant differences in prior laparoscopic and simulation training experience between the groups. Compared with the junior group, a higher proportion of intermediate participants had trained using animal models (*p* = .001), VR models (*p* = .003), and had served as first assistant in laparoscopic gastrectomy procedures (*P* < .001).

**Table 1:** Baseline characteristics of the three groups of trainees

	Intermediate group (*n* = 10)	Junior group (*n* = 10)	*P* value
Age (years), mean	32.20 ± 2.20	25.90 ± 0.74	< 0.001
Sex, n (%)			
Male	10 (100.00)	9 (90.00)	1.000
Female	0 (0.00)	1 (10.00)	
Clinical experience (years), mean	6.90 ± 1.10	2.80 ± 0.63	< 0.001
Hand dominance, n (%)			
Right hand	10 (100.00)	8 (80.00)	0.474
Left hand	0 (0.00)	2 (20.00)	
Cases of LG as first assistant, n (%)			
0	2 (20.00)	9 (90.00)	< 0.001
1–20	5 (50.00)	1 (10.00)	
More than 20	3 (30.00)	0 (0.00)	
Training experience, n (%)			
Animal model	3 (30.00)	0 (0.00)	0.001
VR model	7 (80.00)	0 (0.00)	0.003
Silicone suture pads	10 (100.00)	10 (100.00)	-
3D-Printed model	0 (0.00)	0 (0.00)	-

*LG *Laparoscopic gastrectomy, *VR *Virtual reality, *3D-Printed *three-dimensional printed

### The face validity of model

We asked ten expert surgeons from the senior group to evaluate the face validity of the model. As summarized in Table 2, the model received the maximum score (5.0) in the categories of overall impression, anatomical size, realism, workspace simulation, tearing tendency, and suture penetration resistance. The texture of the model, however, received a lower rating (3.70 ± 0.82), suggesting a potential area for future improvement. Excellent inter-rater reliability was confirmed (Kappa = 0.87, 95% CI: 0.82–0.92, *p* < .001).

**Table 2:** Evaluation of the model facial validity

	Mean ± SD
Overall impression	5.00 ± 0.00
Size	5.00 ± 0.00
Texture	3.70 ± 0.82
Realism	5.00 ± 0.00
Workspace	5.00 ± 0.00
Tearing tendency	5.00 ± 0.00
Suture penetration resistance	5.00 ± 0.00

*SD *Standard deviation

### The content validity of model

The model also demonstrated strong content validity, as evaluated by the experts, with all items receiving mean scores above 4.0 (Table 3). Experts strongly agreed that the model effectively improved technical skills, enhanced confidence, accelerated the learning process, and reduced patient risk. They also confirmed that the model accurately simulated critical surgical scenarios and endorsed its integration into structured LDSRS training programs.

**Table 3:** Evaluation of the model content validity

	Mean ± SD
The model was easy to handle.	4.60 ± 0.52
The operation on the model was similar to an clinical actual case.	4.70 ± 0.48
Practicing with the model could improve surgeons’ skills in performing LDSRS.	4.60 ± 0.70
Practicing with the model could enhance surgeons’ confidence in performing LDSRS.	4.30 ± 0.67
Practicing with the model could shorten the learning curve for LDSRS.	4.60 ± 0.52
Practicing with the model could reduce risks for patients in real surgery.	4.60 ± 0.52
Practicing with the model provided more educational value than clinical observation alone.	4.30 ± 0.48
I recommend using this model for training the LDSRS.	5.00 ± 0.00

*LDSRS *Laparoscopic duodenal stump reinforcement suture; SD: Standard deviation

### Operative performance in initial training

**Fig. 4:**
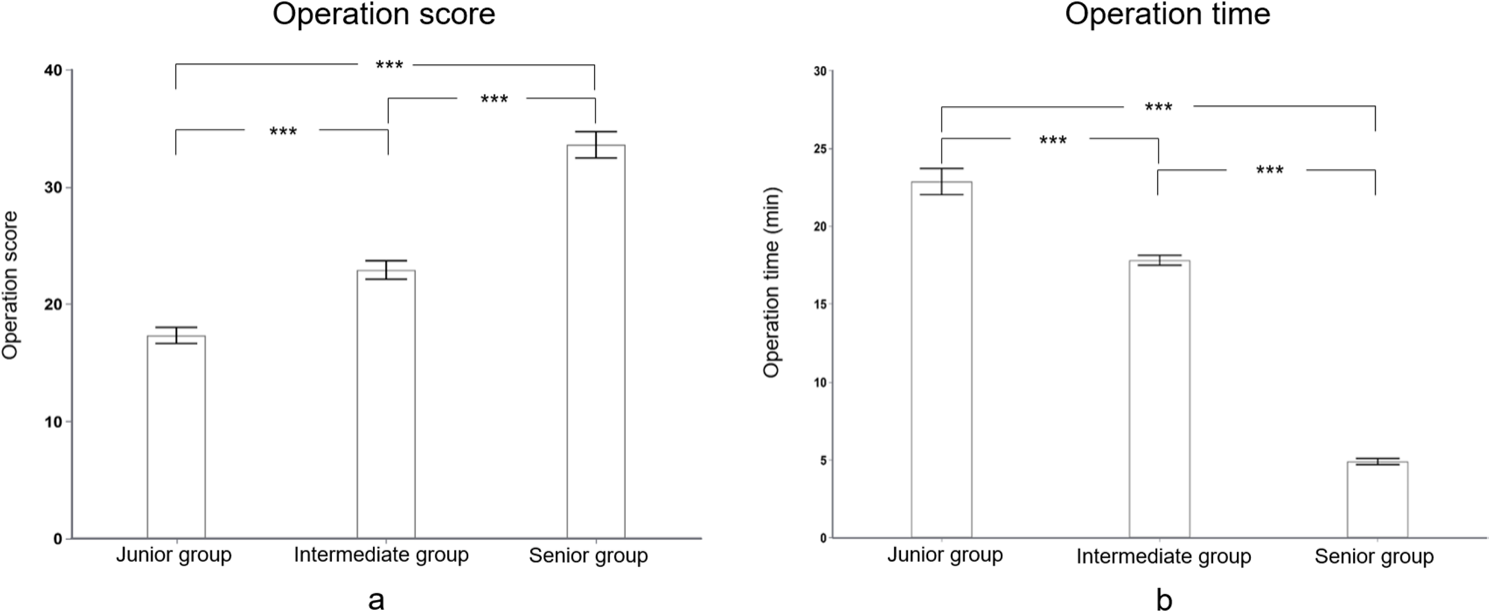
The operation performances in the initial training session. **a** The operation score of the senior group was significantly higher than either that of the intermediate or junior groups (**b**) The operation time of the senior group was significantly shorter than either that of the intermediate or junior groups. ***p < .001

The model demonstrates strong construct validity, effectively discriminating between surgeons of varying clinical experience. Table 4; Fig. 4a present the operative scores for participants in the initial training session. The scores of the junior and intermediate groups were significantly lower than those of the senior group (17.35 ± 0.69 vs. 22.90 ± 0.79 vs. 33.60 ± 1.13, all *p* < .001). Similarly, operative times differed significantly among the three groups. As shown in Table 4; Fig. 4b, the operative time for the junior and intermediate groups was significantly longer than that for the senior group (22.87 ± 0.84 min vs. 17.81 ± 0.32 min vs. 4.90 ± 0.20 min, all *p* < .001).

**Table 4:** The operation scores and operation times of the three groups

	Junior group	Intermediate group	Senior group	*P* value
Initial training session				
Operation score, mean	17.35 ± 0.69	22.90 ± 0.79	33.6 ± 1.13	< 0.001
Operation time (min), mean	22.87 ± 0.84	17.81 ± 0.32	4.9 ± 0.20	< 0.001
10th training session				
Operation score, mean	26.03 ± 0.40	28.53 ± 0.53	33.6 ± 1.13	< 0.001
Operation time (min), mean	8.36 ± 0.20	7.35 ± 0.20	4.9 ± 0.20	< 0.001

### Operative Performance in Repeated Training

**Fig. 5:**
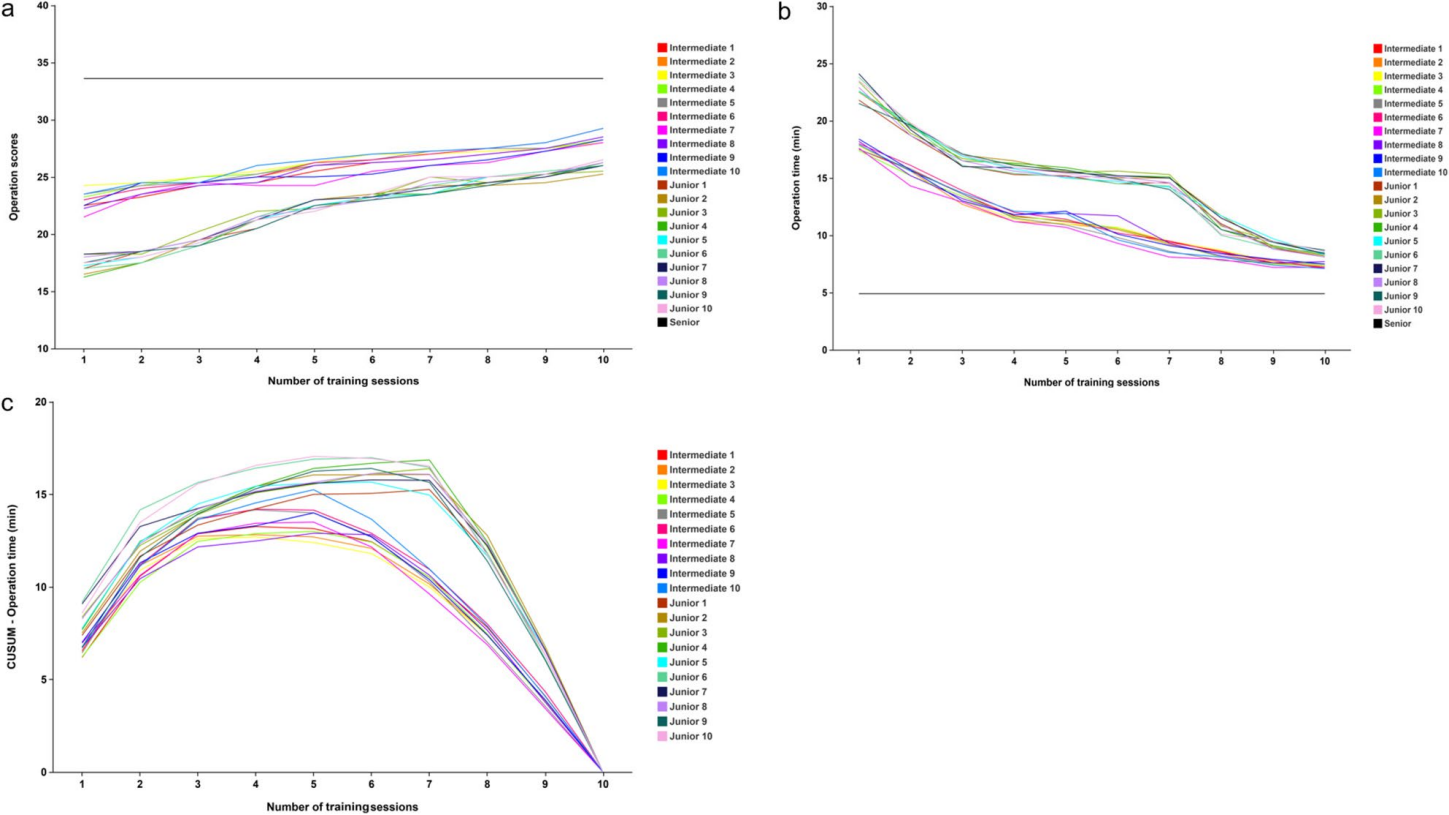
(**a**) The operation score curve of the twenty trainees from intermediate and junior groups (**b**) The operation time curve of the twenty trainees from intermediate and junior groups (**c**) The learning curve of the twenty trainees from intermediate and junior groups

**Fig. 6:**
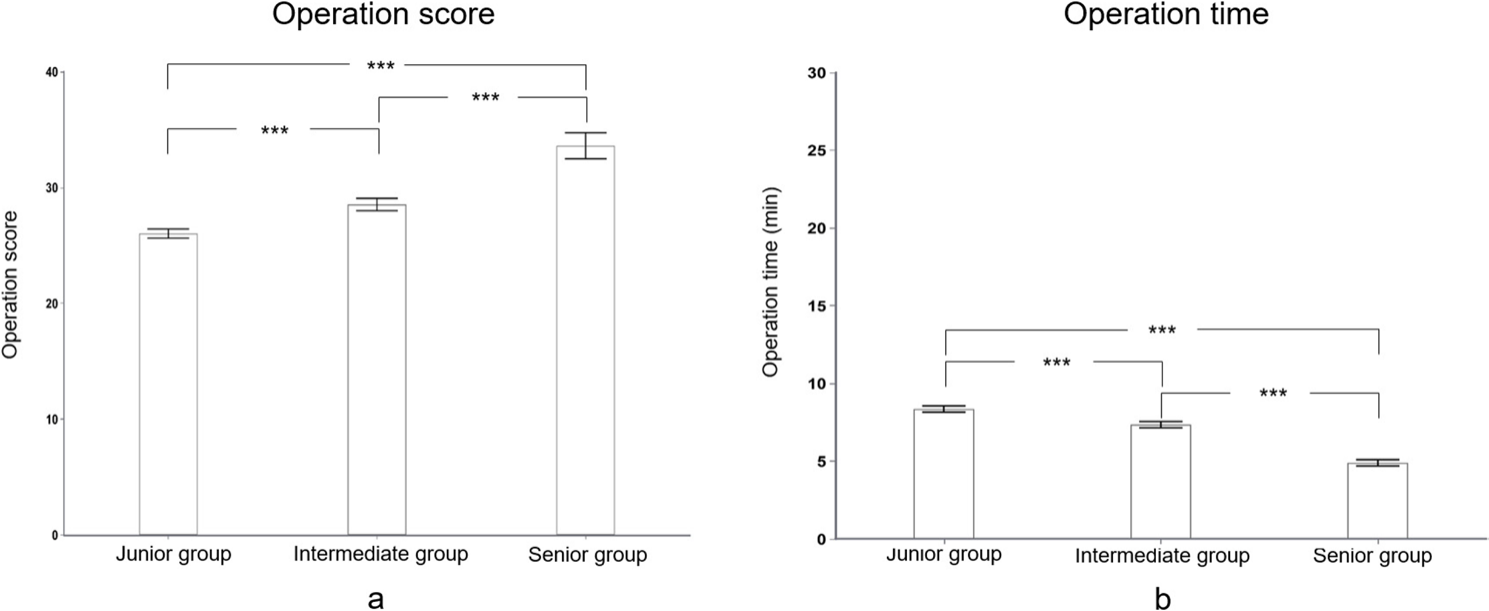
The operation performances in the tenth initial training session. **a **The operation score of the senior group was significantly higher than either that of the intermediate or junior groups (**b**) The operation time of the senior group was significantly shorter than either that of the intermediate or junior groups. ***p < .001

We plotted the operative scores of the twenty trainees across the ten training sessions on a line graph, which demonstrated a consistent upward trend as training progressed (Fig. 5a). The operative scores for the junior and intermediate groups in the final training session are presented in Table 4; Fig. 6. Results revealed that despite improvement, the junior and intermediate groups still had significantly lower average scores than the senior group (26.03 ± 0.40 vs. 28.53 ± 0.53 vs. 33.60 ± 1.13, all *p* < .001).

Figure 5b displays the operative time curve for twenty trainees over the ten training sessions. As training progressed, operative time gradually decreased and eventually stabilized after a certain number of sessions. We also compared the final-session operative times across the three groups (Table 4 and Supplementary Fig. 1b). The results showed that both the junior and intermediate groups still required significantly longer operative times than the senior group (8.36 ± 0.20 min vs. 7.35 ± 0.20 min vs. 4.90 ± 0.20 min, all *p* < .001).

### Learning curve assessment

Figure 5c illustrates the CUSUM learning curve for the twenty trainees. Among the ten trainees in the junior group, the turning points in their learning curves occurred during the 6th or 7th training session. In contrast, the ten trainees in the intermediate group reached this proficiency point earlier, typically by the 4th or 5th session.

## Discussion

This study developed and validated a safe, cost-effective 3D-printed mold-based simulation model for LDSRS. The model’s efficacy aligns with the expanding application of 3D printing in medicine [31–33]. This study demonstrates that the model possesses excellent face, content, and construct validity, accurately replicating the critical anatomy and biomechanical properties of the duodenal stump. The validity evidence presented in this study—encompassing expert evaluations (content and response process), discrimination between experience levels (relations to other variables), and internal consistency of metrics—contributes to a modern argument for validity [18]. Furthermore, repeated training with this model significantly improved trainees’ operative performance, effectively compressing the early, steep phase of the learning curve.

The authenticity of the model reflects the fidelity of simulated training compared to clinical reality, serving as a crucial indicator for assessing the model’s validity [34, 35]. Given that the accurate assessment of authenticity requires extensive clinical experience, evaluation in this study was performed exclusively by ten expert surgeons (senior group). Similar to previously published 3D-printed models [15, 16, 19], our model demonstrates high face validity and content validity. Apart from texture (3.70 ± 0.82), experts rated the model with perfect score (5.00 ± 0.00) in all other aspects, indicating strong endorsement of its authenticity. Additionally, experts unanimously agreed that training with this model effectively enhances surgical skills, boosts self-confidence, reduces surgical risks, shortens the learning curve, and endorsed its widespread application in LDSRS training.

In this study, we utilized soft silicone material to fabricate hollow organ models, including the stomach and duodenum, which formed the core components of the training model. The parenchymal organ models, such as the liver, gallbladder, and pancreas, were also integrated into the simulator to replicate the confined spaces encountered during actual LDSRS procedure. Additionally, throughout the fabrication process, we continuously adjusted the silicone oil ratio in the silicone gel to modify the of the model’s mechanical properties. In previous studies, we used ultrasound elastography to measure the maximum stress (0.74 MPa) and hardness (1.95 m/s) of our duodenum model, respectively [16, 19]. These parameters closely approximate the reported biomechanical properties of a normal human small intestine (maximum stress of 0.9 MPa [36] and a hardness of 1.42 m/s [37]), ensuring that the suturing sensation of our model resembled that of a real duodenum. This high biomechanical fidelity may facilitate the transfer of skills acquired on the simulator to the operating room, a premise that requires validation in future clinical studies.​​.

Apart from authenticity, assessing surgeons’ technical skills is crucial in surgical simulation training, as it positively influences patient prognosis and treatment outcomes [38]. This study evaluated the construct validity of the model by comparing operation scores and time between the intermediate and junior groups with the senior group. Consistent with previous studies about 3D-printed training models, our model demonstrated strong discriminative ability [15, 16, 39]. In initial training sessions, the junior and intermediate groups achieved significantly longer operation times and lower operation scores compared to the senior group, suggesting that the model can effectively distinguish between surgeons with varying levels of clinical experience. During the repeated training phase, operation scores for all trainees exhibited a gradual upward trend, while the operation times showed a gradual downward trend. These findings indicate that the model can effectively enhance surgeons’ operation proficiency, validating the effectiveness of simulation training. Furthermore, we found that even after ten training sessions, differences in operation scores and times remained between the intermediate and junior groups compared to the senior group. The persistent performance gap underscores the extended practice required and highlights that proficiency benchmarks, rather than training duration alone, should guide readiness assessment. This further highlights the clinical applicability and value of the model.

The learning curve, based on operation time, reflects the rate of skill or knowledge acquisition over a specified period and is commonly used to assess the number of training sessions required to achieve proficiency in surgical techniques [39, 40]. In this study, the CUSUM learning curve revealed that operation times for the intermediate and junior groups reached turning points after 4th to 5th and 6th to 7th training sessions, respectively. The turning point in the CUSUM curve signifies a trend transition, suggesting that surgeons with more clinical experience may achieve a skilled stage with fewer training sessions. Although the operation time for the junior and intermediate groups remained significantly longer than those of the senior group after ten training sessions, we believe that with continued training, their operation time will decrease further and may eventually reach the mastery stage of the senior group.

The cost of simulation training is also a significant consideration, even though its primary aim is to enhance learning. Our model demonstrated exceptional cost-effectiveness, with a manufacturing cost of approximately 500 US dollars, which is lower than most existing models. This cost advantage can be attributed to several factors. First, during the model’s production process, we employed an indirect printing method: molds were printed using a 3D printer, and the models were then fabricated by casting using molds. This approach allowed for multiple mold reuses, regardless of material changes, significantly increasing productivity compared to direct model printing. As production volumes increase in the future, the per-unit cost of the model is expected to decrease further. Second, our model was constructed from soft silicone materials, which are highly elastic and chemically inert, contributing to a longer lifespan for the model [41, 42]. Third, to ensure model reusability, easily consumable components such as the stomach and duodenum models were designed to be modular and replaceable. These features significantly reduce costs, creating an affordable training tool that enhances surgical education accessibility in under-resourced and remote regions.

This study has several limitations. First, the absence of a control group limits our ability to definitively conclude that the developed simulation model is superior to existing training methods. The primary aim of this pilot study was to establish the model’s feasibility, face, content validity, and its ability to distinguish between different experience levels. Comparative effectiveness studies with control groups are a necessary and valuable direction for future research. Second, and most importantly, we presently lack evidence that skills acquired on the model translate to improved patient outcomes in the operating room. Although current validity evidence is robust, future randomized trials are mandated to confirm that this model training translates to superior operative performance and reduced DSF rates in real patients with GC. Third, the questionnaire used to assess face and content validity, along with the modified laparoscopic performance scoresheet, were not formally validated instruments. While they were developed based on established surgical assessment principles and expert input, the lack of prior validation may limit the generalizability of our subjective findings. Future studies would benefit from employing standardized, validated assessment tools to further corroborate these results.

## Conclusion

The 3D-printed mold-based silicone model demonstrated high face and content validity, indicating strong authenticity and perceived educational utility. Its construct validity suggests it is an effective tool for distinguishing between different skill levels within the simulated environment. These findings support its integration into structured simulation-based training curricula for LDSRS as a valid and effective training tool. Future studies are required to evaluate the transfer of acquired skills to the operating room and any potential impact on clinical outcomes.

## Data Availability

The datasets generated and/or analysed during the current study are available from the corresponding author on reasonable request.
